# A stacked cavity-backed metamaterial antenna for confined hyperthermia of subcutaneous fat in ISM band

**DOI:** 10.3389/fmedt.2026.1768771

**Published:** 2026-06-10

**Authors:** Dong-Min Lee, Yun-Jae Jung, Ikhwan Kim, Kyujin Cho, Qingzhou Wang, Seongwoog Oh, Eun-Seong Kim, Nam-Young Kim

**Affiliations:** 1 RFIC Bio Center, Kwangwoon University, Seoul, Republic of Korea; 2 Department Electronic Engineering, Kwangwoon University, Seoul, Republic of Korea; 3 R&D Center, APR Co., Ltd., Seoul, Republic of Korea; 4 School of Electrical and Computer Engineering, University of Seoul, Seoul, Republic of Korea; 5 Department of Molecular Medicine and Biopharmaceutical Sciences, Seoul National University, Seoul, Republic of Korea; 6 Department of Medical and Digital Engineering, College of Engineering, Hanyang University, Seoul, Republic of Korea

**Keywords:** deep tissue targeting, hyperthermia, metamaterial antenna, near-field control, on-Body matching

## Abstract

**Introduction:**

Hyperthermia has emerged as a versatile modality in oncology, rehabilitation, and aesthetic medicine, yet conventional RF devices often suffer from insufficient penetration and collateral epidermal heating. This study proposes a miniaturized stacked cavity-backed antenna operating in the 2.4-2.5 GHz ISM band for depth-confined heating of facial subcutaneous fat.

**Methods:**

By leveraging the strong dielectric contrast at the skin-fat boundary, the design enhances the normal electric-field component in adipose tissue to promote preferential power deposition without active epidermal cooling. Electromagnetic simulations quantify field and SAR confinement, and multiphysics thermal simulations evaluate short-pulse therapeutic operation. The methodology is validated through ex vivo porcine experiments and a preliminary in vivo human feasibility test (10 W, surface thermography).

**Results:**

The results confirm reproducible superficial warming at the target depth without adverse skin reactions. The study successfully demonstrates single-element hardware feasibility and effective depth-selective energy confinement within the subcutaneous fat layer.

**Conclusion:**

This research establishes the fundamental hardware capability for targeted facial hyperthermia. However, macroscopic treatment-uniformity protocols and long-term clinical efficacy remain topics for future clinical studies.

## 1 Introduction

Hyperthermia, the therapeutic application of controlled heat to biological tissues, has been extensively studied across various medical fields, including oncology, dermatology, and physiotherapy (1–4). Clinically, hyperthermia has been utilized to enhance tumor sensitivity to radiotherapy and chemotherapy, to promote wound healing, and to improve local blood circulation in musculoskeletal disorders (5–9). In recent years, dermatologic and aesthetic applications of hyperthermia have gained remarkable attention, driven by the growing demand for non-invasive procedures for skin rejuvenation and body contouring (10, 11). Market analyses indicate a continuous annual growth of more than 10% in the global aesthetic device sector, with radiofrequency (RF)-based hyperthermia systems being one of the most rapidly expanding segments due to their clinical safety, minimal downtime, and reproducible results (12, 13).

In dermatology and cosmetic medicine, various surface-focused RF and infrared (IR) hyperthermia devices have been employed for skin tightening, wrinkle reduction, and cellulite treatment by inducing collagen denaturation and stimulating neocollagenesis within the dermis (14, 15). More recently, many of these devices have been designed to target the subcutaneous fat layer to achieve volumetric skin tightening or localized fat reduction. However, a fundamental limitation persists most existing RF and IR systems lack sufficient field confinement and depth targeting, resulting in unwanted heating of superficial layers such as the epidermis and dermis along with the targeted fat tissue (16, 17). This non-selective heating not only reduces energy efficiency but also increases the risk of pain, burns, and uneven thermal profiles within the treatment area (18, 19).

In contrast, selective hyperthermic targeting of the subcutaneous fat layer provides distinct therapeutic and aesthetic advantages (20). The subcutaneous adipose tissue acts as both a primary energy absorber and a biologically responsive medium; maintaining the temperature within 42–45 °C can induce adipocyte thermolysis or apoptosis, leading to gradual fat volume reduction and improved contour without damaging overlying tissues (21–24). Achieving this targeting, however, requires precise control over electromagnetic field penetration and localized energy deposition, which are strongly influenced by the dielectric properties of skin, fat, and muscle (1, 25, 26).

At ISM band frequencies (e.g., 2.4–2.5 GHz), the dielectric contrast among these tissue layers allows engineered antennas to be designed for depth-specific energy confinement (2). While recent advancements have successfully demonstrated that multisource radiofrequency systems (15) and compact directive microwave radiators can achieve focused field distributions to selectively heat targeted deep-tissue regions, these multi-element or complex systems typically require sophisticated phase-control networks and occupy a larger physical footprint, limiting their practicality for highly compact aesthetic handpieces.

Conversely, most conventional compact single-element applicators, such as lower-frequency capacitive-resistive RF devices (24, 27), still suffer from broad near-field distributions that inadvertently heat multiple superficial tissue layers simultaneously. Furthermore, while highly miniaturized monopole antennas have been developed for specific minimally invasive interventions such as catheters, securing sufficient high-power durability-the structural and thermal ability to safely handle therapeutic input power (e.g., 50–100 W) without suffering from electrical breakdown-within a single compact footprint for rapid, non-invasive hyperthermia remains a significant engineering challenge. To overcome these collective limitations, a highly durable, compact single-element antenna capable of concentrating high-power electromagnetic energy exclusively within the subcutaneous fat layer is strictly essential (28).

**Figure 1:**
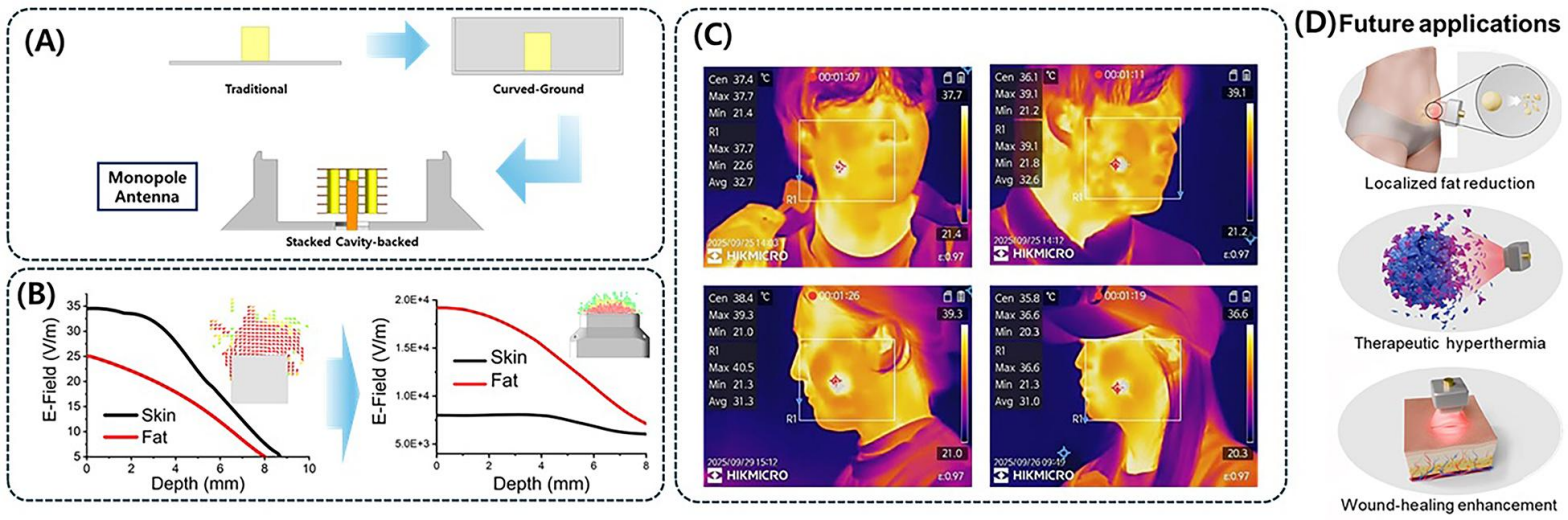
Conception of the proposed stacked cavity-backed monopole antenna for Fat-targeted hyperthermia. **(A)** The Process of Change from Traditional Monopole Antenna to The Proposed Stacked Cavity-backed Monopole Antenna, **(B)** The Difference Between E-field Strength and Vector that Enables Fat Layer Targeting of Typical Waveguide Antenna and The Proposed Monopole Antenna, **(C)** RF Hyperthermia Performance Measured by Thermal Imaging Cameras **(D)** Future Application Fields.

Therefore, this study proposes and optimizes a stacked cavity-backed monopole antenna operating in the ISM band specifically engineered for non-invasive, fat-layer-targeting hyperthermic stimulation (29, 30). As conceptualized in Figure 1, the intended clinical utility of this targeted approach spans two primary domains. First, for cosmetic applications, the objective is localized fat reduction and skin tightening; by selectively elevating and maintaining the subcutaneous adipose tissue at 42–45 °C, the device safely induces adipocyte apoptosis and stimulates dermal collagen remodeling. Second, for therapeutic hyperthermia, the device functions as targeted deep-tissue physiotherapy, aiming to increase localized blood perfusion and metabolic activity to accelerate wound healing and relieve superficial musculoskeletal pain.

Crucially, to physically ensure epidermal safety during these procedures, the proposed structure is designed to achieve localized energy confinement by controlling near-field patterns and impedance matching. By leveraging the severe dielectric contrast between tissue layers, the normal electric field vector is inherently concentrated exclusively within the low-permittivity fat layer. This mechanism fundamentally bypasses excessive thermal accumulation in the highly conductive superficial skin, effectively preventing contact burns while maximizing power deposition within the adipose layer. Through comprehensive electromagnetic simulation, SAR (specific absorption rate) profiling, and thermal modeling, this work demonstrates the feasibility of selective subcutaneous heating, providing a foundational design framework for next-generation aesthetic and therapeutic hyperthermia systems with inherently improved safety and precision.

## 2 Materials and methods

### 2.1 Antenna design

The proposed antenna was designed with a focus on compactness, efficient penetration into biological tissues, and selective stimulation of subcutaneous fat layers (21–23). The overall dimensions were determined as 24 mm × 24 mm × 21.6 mm. This specific dimensional optimization was fundamentally driven by the anatomical requirements of the primary target application: targeted-depth hyperthermia of facial subcutaneous fat. Because facial structures (e.g., cheeks and jawline) are highly non-planar and feature complex contours, this compact 24 mm × 24 mm footprint was engineered to maintain stable, conformal contact against the curved skin-thereby minimizing energy-scattering air gaps-while ensuring sufficient electromagnetic penetration and practical handling (31). If the antenna size were excessively reduced, coverage would become impractically limited. Conversely, an oversized structure would fail to conform to facial curves, weakening localized energy concentration. Regarding practical clinical translation, it is important to note that establishing protocols for macroscopic treatment uniformity is not the main focus of this foundational hardware study. While experimental validations may utilize prolonged stationary exposures (e.g., 1 min) purely to observe maximum thermal accumulation limits, the actual clinical application is expected to employ a rapid “pulse-and-move” (or stamping) technique. The practitioner would deliver a short RF pulse (e.g., 1–2 s) to a localized site before immediately repositioning to an adjacent area. Establishing precise clinical guidelines to ensure uniform treatment across the entire facial surface falls outside the current scope and remains a key objective for future clinical research. Conversely, an oversized structure would weaken the concentration of energy. Thus, the chosen dimensions represent a balance between efficiency and operability. The operating frequency was selected based on the trade-off between penetration depth and antenna miniaturization. At higher frequencies, penetration into tissue becomes limited, whereas at lower frequencies, antenna size grows too large to achieve precise concentration. To address these constraints, the 2.4–2.5 GHz industrial, scientific, and medical (ISM) band was chosen, which not only offers appropriate penetration depth but also allows for a compact antenna geometry. In addition, this frequency band is widely allocated for biomedical and therapeutic use, making it suitable for hyperthermia applications.

The choice of substrate material was critical to both miniaturization and high-power tolerance. A substrate with high permittivity was necessary to reduce antenna size, while low dielectric loss and high thermal stability were required for reliable operation under elevated power levels. RF-10 $\left(\varepsilon_{r}=10.2,\;\mu =1,\;\tan\;\delta =0.0025\right)$ was selected due to its excellent electrical and thermal performance, as well as its commercial availability, ensuring that the design could be practically implemented (32).

Structurally, the antenna was based on a monopole configuration, which inherently provides good penetration characteristics. However, since monopole radiate omnidirectionally, they are not inherently suitable for local targeted fat-layer heating. To overcome this, the ground plane was folded into a cavity-backed structure, transforming the radiation into a more directional pattern toward the tissue (33). Additionally, a stacked multilayer dielectric approach was employed using standard multilayer printed circuit board (PCB) manufacturing and lamination process to enhance resonance properties and ensure robust physical fabrication. Specifically, the internal radiating structure consists of 7 stacked layers of RF-10 substrate, with each individual layer having a thickness of 40 mil, resulting in core radiator dimensions of 8.5 mm × 8.5 mm. To permanently bond these stacked dielectric layers during the lamination process, a standard RF-compatible adhesion material (FR4 Prepreg with a thickness of 70 um) was utilized between the substrate interfaces. The surrounding metallic cavity is fully filled to maximize the effective permittivity and ensure mechanical rigidity.

**Figure 2:**
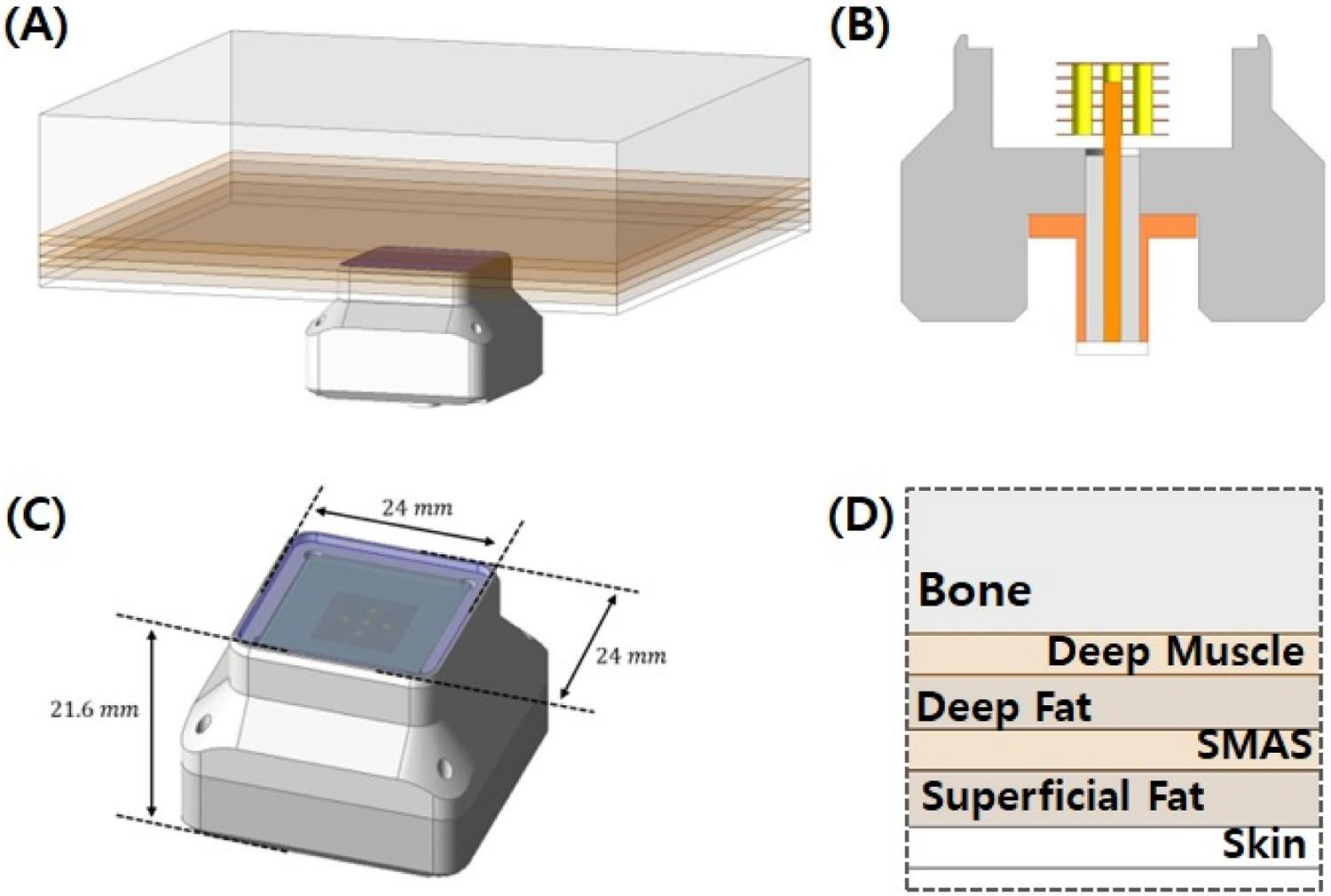
**(A)** Overview of stacked cavity-backed monopole antenna with flat phantom and **(B)** structure of conductors for stacked cavity-backed monopole antenna and **(C)** dimension of stacked cavity-backed monopole antenna, and **(D)** structure of designed facial flat phantom.

For the vertical pattern implementation, the stacked conductive monopole layers were vertically interconnected using a dense array of plated through-hole (PTH) vias. It is important to note that while discrete vertical vias physically cannot form a perfectly solid continuous metal block due to standard via-density manufacturing constraints, this optimized high-density via array guarantees robust and continuous RF current flow across the entire interconnection area at the 2.45 GHz operating frequency. Any minor physical discontinuities between the vias are electromagnetically compensated by strong high-frequency capacitive coupling between the closely stacked conductive planes. Consequently, this dense interconnection ensures that the entire stacked assembly electrically functions as a unified, thick volumetric monopole radiator, reliably achieving stable current distribution and robust broadband impedance matching. To clearly illustrate these internal fabrication details, high-resolution cross-sectional and exploded views depicting the layer stack, adhesion interfaces, and via structures are provided in the Figure 2.

The final geometry was optimized so that the S11 parameter remained below −10 dB across the ISM band (2.4–2.5 GHz). Furthermore, the antenna was confirmed to remain non-resonant in free-space conditions, which enhances safety by preventing unnecessary radiation exposure when not coupled to tissue. Figure 2A shows overview of the antenna matching while attached to the designed flat phantom.

Finally, the performance of the proposed design was compared with commercial monopole antennas operating at 2.4–2.5 GHz, which typically measure around 24 × 24 × 21.6 (mm). The new structure achieved notable miniaturization by combining a cavity-backed ground and high-permittivity substrate. Figures 2B,C shows cavity-backed ground, and radiator made by stacked conductors. Simulation results further showed that, unlike conventional monopoles which concentrate energy within the skin layer, the proposed antenna enabled deeper penetration into subcutaneous fat with higher specific absorption rate (SAR) values in the fat layer. This demonstrates the antenna's ability to provide targeted hyperthermia for both cosmetic and medical applications.

### 2.2 Tissue modeling

To accurately evaluate the electromagnetic energy delivery and thermal response of the proposed antenna, a six-layer human facial tissue model was developed. The model comprised, from surface to depth, skin, superficial fat, superficial musculoaponeurotic system (SMAS) muscle, deep fat, deep muscle, and bone, replicating the characteristic anatomical structure of the human face. Facial tissue was selected as the reference because of its relatively thin and compositionally complex layers, which make it particularly responsive to radiofrequency (RF) penetration and localized heating.

Figure 2D shows the structure of the designed facial flat phantom. In the facial region, particularly along the cheek, the soft tissue architecture generally consists of the skin, subcutaneous fat, superficial musculo-aponeurotic system (SMAS), retaining ligaments, and deep fascial layer. Each layer was modeled based on experimentally reported biophysical data of facial tissues at ISM-band frequencies, including physical thickness, dielectric permittivity, and thermal conductivity. (Version 4.1, 2022) The differences in dielectric constant and electrical conductivity among these layers were key factors for determining the target heating region. In particular, the skin and muscle layers exhibit relatively high permittivity and conductivity, whereas the fat layers demonstrate significantly lower values, enabling targeted electromagnetic energy deposition within the adipose tissue. Table 1 shows the physical and dielectric properties assigned to each layer in the designed flat-type facial phantom model.

**Table 1:** Characteristice of designed facial phantom.

Element	Thickness	Permitivity *ε*	Permeability *µ*	Conductivity *σ* (siemens/m)	Loss tangent tan *δ*	Mass density (kg/m^3^)
Skin	1.5 mm	38	1	1.46	0.281	1,109
Superficial fat	2 mm	10.8	1	0.268	0.182	911
SMAS muscle	1.5 mm	52.7	1	1.74	0.242	1,090.4
Deep fat	2 mm	10.8	1	0.268	0.182	911
Deep muscle	1.5 mm	52.7	1	1.74	0.242	1,090.4
Bone	20 mm	11.4	1	0.394	0.254	1,908

The antenna was placed in direct contact with the skin surface to simulate practical coupling and energy transfer behavior. Specific absorption rate (SAR) distributions were extracted to quantify localized power deposition, and E-field profiles were analyzed to verify confinement of electromagnetic energy within the subcutaneous fat region. Furthermore, input power was varied to assess the controllability of temperature elevation across tissue interfaces, while free-space resonance verification ensured operational safety in non-contact conditions. Together, this modeling and validation framework provided a realistic and reproducible foundation for evaluating the antenna's selective heating capability in facial hyperthermia applications.

### 2.3 Thermal analysis

Thermal response was evaluated through coupled electromagnetic-thermal simulations and experimental validation. The antenna design and electromagnetic field distribution were first analyzed using ANSYS HFSS, a finite-element-based (FEM) solver, to calculate the specific absorption rate (SAR) and assess power deposition within the multilayer facial tissue model (30). Subsequently, Sim4Life (ZMT Zurich MedTech AG) was employed to conduct multiphysics simulations integrating both electromagnetic and thermal analyses using multiple high-resolution human anatomical models (34, 35). These models represented different physiological variations, including male and female subjects, variable facial fat thickness, and diverse skin-muscle geometries, enabling robust verification of the antenna's heating performance under anatomically realistic and heterogeneous conditions. All multiphysics simulations were implemented using a coupled electromagnetic–thermal workflow. The transient temperature evolution was computed using Pennes’ Bioheat Transfer Equation, accounting for heat conduction, electromagnetic heating (SAR), and physiological cooling mechanisms including blood perfusion and metabolic heat generation. All tissue-specific thermal and physiological properties were assigned based on the IT'IS Foundation Tissue Properties Database (Version 4.1). At the skin–air interface, a convective heat transfer boundary condition was applied assuming an ambient temperature of 37 °C, and convective heat transfer coefficients were assigned to reflect realistic clinical environments. This approach provided a more comprehensive assessment of energy penetration, field confinement, and selective heating reliability across individual anatomical differences.

A uniform input power of 10 W was applied to evaluate the antenna's hyperthermic performance. Temperature rise was monitored in each tissue layer to verify selective heating of the subcutaneous fat regions while minimizing unwanted surface heating. The correlation between SAR distribution and thermal elevation was analyzed to confirm the thermal confinement effect predicted by the electromagnetic field pattern.

For experimental verification, *ex vivo* tests were performed using pig tissue, which exhibits dielectric and thermal properties similar to human fat and muscle (36, 37). The antenna was placed in direct contact with the tissue surface, and temperature changes were recorded over 1 min of continuous RF exposure under identical power conditions. Although some deviations from the simulated results were observed due to uncontrolled biological variability, the overall heating pattern was consistent with the simulation trend. Specifically, both the human tissue model and pig tissue experiments confirmed localized heating behavior, demonstrating that the antenna can effectively concentrate thermal energy within the subcutaneous fat layer while maintaining minimal temperature rise in the superficial skin region.

**Figure 3:**
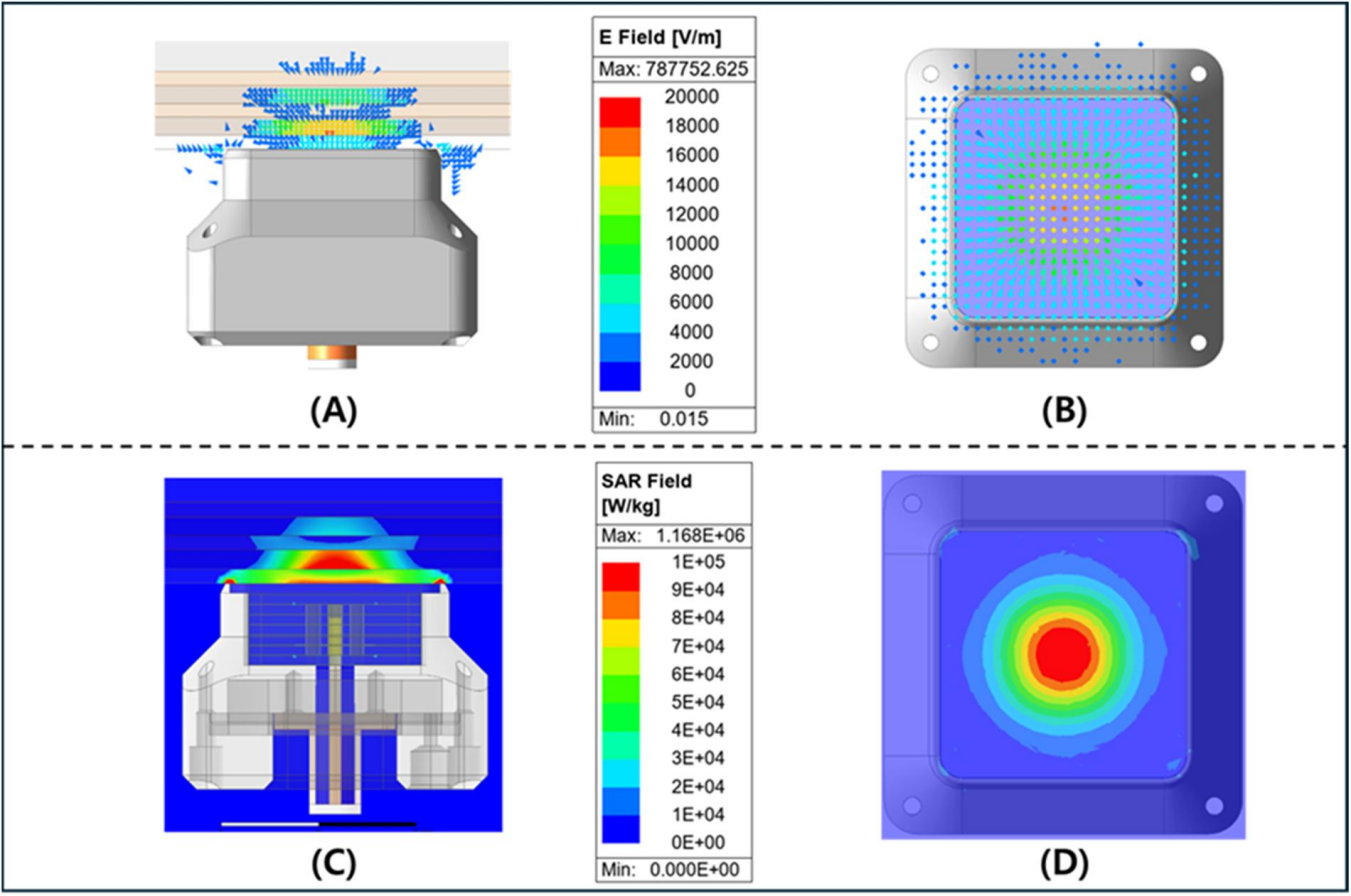
Vector E field distribution in **(A)** vertical section and **(B)** fat layer section, and SAR distribution in **(C)** vertical section and **(D)** fat layer section.

The spatial distribution of heating was further analyzed by examining the correlation between SAR and temperature rise across the tissue layers (43). Specific absorption rate (SAR) was computed as the time-average power absorbed per unit mass. In Ansys HFSS, local SAR is calculated at each mesh location as

$$\mathrm{SAR}=\frac{\sigma {\left|E\right|}^{2}}{2\rho }$$ (1)

where |E| is the magnitude of the electric field (V/m), ρ is the mass density (kg/m^3^), and σ is the effective conductivity defined by the solver (including dielectric-loss contribution when loss tangent is used). We report both (i) high-resolution local SAR maps to visualize boundary-localized energy confinement and (ii) peak spatial-average SAR (psSAR) over 1 g and 10 g to enable standardized comparison, computed using an IEC/IEEE-compliant averaging procedure. All SAR metrics are normalized to the specified net input power at the antenna feed. Because SAR is directly proportional to the square of the local electric field, its spatial pattern follows the field intensity distribution within the tissue. The proposed monopolar antenna generates an omnidirectional electromagnetic field predominantly characterized by a normal (perpendicular) electric field component at the tissue interface (21–23). According to Maxwell's boundary conditions, the tangential and normal components of the electric field satisfy:

$$E_{1t}=E_{2t},\;\mathrm{and}\;\varepsilon_{1}E_{1n}=\varepsilon_{2}E_{2n}$$ (2)

where ε denotes the permittivity of each medium. Since the heating interaction is dominated by the normal field component, the local field strength becomes inversely related to permittivity at the boundary according to (Equation 2). Although the skin is highly conductive $\left(\sigma_{\mathrm{skin}}=1.46\;S/m\right)$, its thin physical profile (1.5 mm) compared to the penetration depth (∼15–20 mm at 2.45 GHz) limits absolute attenuation. At the skin-fat interface, the normal E-field component in the fat layer is amplified by a factor of ∼3.5 $\left(\varepsilon_{\mathrm{skin}}/\varepsilon_{\mathrm{fat}}\approx 38/10.8\right)$ due to boundary conditions. Since $\mathrm{SAR}\propto \sigma |E{|}^{2}$, the squared E-field enhancement (∼12.25×) physically overrides the skin's higher conductivity (∼5.4× greater than fat), effectively concentrating the peak SAR and thermal energy within the subcutaneous fat layer. Consequently, between the skin $\left(\varepsilon_{\mathrm{skin}}=38\right)$ and the subcutaneous fat layer $\left(\varepsilon_{\mathrm{fat}}=10.8\right)$, the lower permittivity of fat produces a stronger electric field amplitude in this region (25). As SAR is proportional to $|E{|}^{2}$ as (Equation 1), this results in selectively higher SAR values and thermal concentration within the subcutaneous fat, while adjacent skin and muscle layers experience weaker field intensity and milder heating. This physical mechanism explains the thermal confine effect consistently observed in both simulations and experiments. Figure 3 shows the E-field and SAR distributions generated using HFSS under static conditions. The reported SAR maps represent un-averaged local point SAR evaluated discretely at each finite element mesh tetrahedron and were normalized to an input power of 100 W delivered at the antenna feed port. While 1 g/10 g spatially averaged SAR is a standard metric for regulatory safety evaluation, applying a 1 g volume average (approximately 1 cm^3^ of tissue) across the extremely thin (1.5 mm) skin–fat boundary would mathematically smooth out and dilute the sharp localized peak E-field enhancement that is central to the proposed near-field confinement mechanism. Therefore, un-averaged local SAR is presented to preserve the highest spatial fidelity at the dielectric interface. Dynamic physiological variables such as blood perfusion and metabolic heat generation were not included in this EM-only analysis and were instead incorporated in the subsequent multiphysics thermal simulations [C95.3, 2021; (38)]. The dominance of the normal E-field vector inherently guides electromagnetic energy to accumulate within the fat layer, enabling selective and efficient subcutaneous hyperthermia under non-invasive operating conditions.

## 3 Result

### 3.1 Experimental validation using pig and human tissue measurements

To experimentally verify the heating characteristics observed in simulations, both *ex vivo* and *in vivo* tests were conducted under identical operating conditions (10 W input power, 1 min exposure).

For the *ex vivo* evaluation, fresh pork tissue was employed because its dielectric and thermal properties closely resemble those of human fat and muscle. The antenna was placed in direct contact with the tissue surface, and embedded wire-type thermocouples were inserted at multiple depths corresponding to the skin layer, subcutaneous fat, SMAS layer, and deep fat layer, enabling simultaneous measurement of temperature variations across different depths.

**Figure 4:**
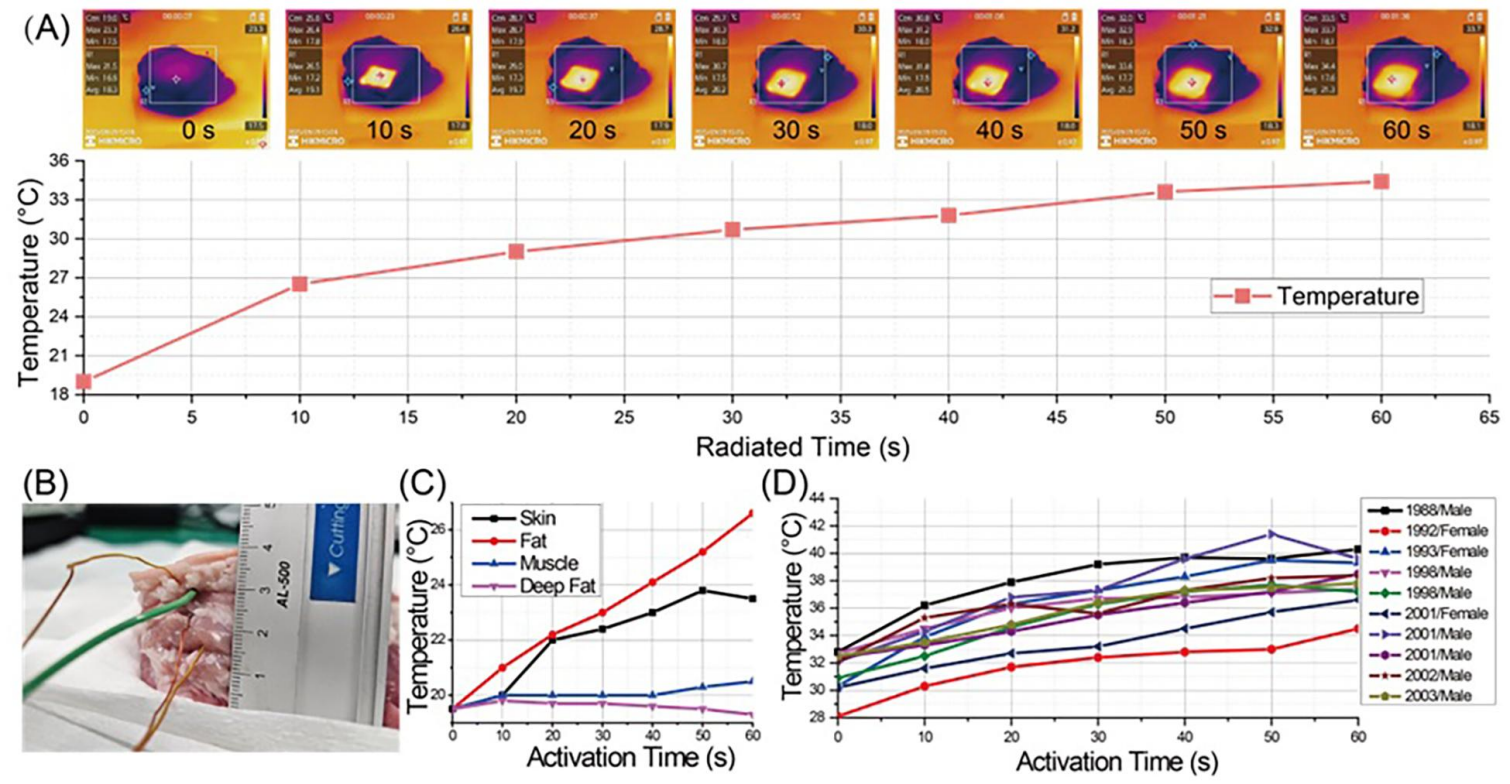
Temperature change in RF exposure measurements. **(A)** The surface temperature of pig tissue with thermal imaging cameras, **(B)** the measurement scene with wire thermometer, **(C)** the temperature changes in each tissue layer, and **(D)** the temperature change of people with thermal imaging cameras.

For the *ex vivo* evaluation, fresh pork tissue was employed. Although the complex permittivity of the specific porcine tissue layers was not independently measured prior to the experiments, this decision was robustly supported by an extensive body of established bioelectro-magnetic literature. Numerous rigorous studies have demonstrated that the dielectric and thermal properties of freshly excised porcine tissue at the 2.45 GHz ISM band are highly consistent and closely resemble those of human fat and muscle, making it a globally accepted and standardized experimental surrogate for evaluating near-field microwave antennas (36, 39). Real-time infrared thermography was used to monitor surface temperature variations. Although the thermal camera primarily captured surface heating, the spatial distribution patterns confirmed that the antenna could induce measurable thermal responses within the targeted region, validating the feasibility of fat-layer heating within safe exposure limits. Figures 4A–C shows the overall thermal response trends obtained from the *ex vivo* pork tissue measurements (40).

In the human tissue experiments, the antenna was placed in direct contact with the center of the cheek. To prioritize absolute patient safety and evaluate fundamental heating feasibility rather than cosmetic efficacy (which typically requires 50–100 W), a conservative input power of 10 W was applied. During the exposure, only surface temperature was monitored using an infrared camera, as direct measurement of deep-layer temperature was clinically impractical. The average surface temperature increase across the ten participants was approximately 5 °C. Importantly, the participants verbally reported a comfortable tactile sensation of localized warming without any thermal discomfort, and post-procedure observations confirmed the complete absence of adverse superficial effects, such as epidermal burns or erythema. Despite variations in baseline temperature and body fat ratio, the experiments demonstrated consistent and reproducible safe heating capability, confirming the antenna's ability to generate uniform warming across individuals. Figure 4D shows the thermal response trend among the ten volunteers during antenna operation.

In pork tissue experiments, two complementary measurement methods were used: infrared thermography and invasive probe thermometry. Figure 4A shows the surface heating trend obtained via thermal imaging. The surface temperature increased rapidly by 7.5 °C within the first 10 s, followed by an average rise of 1.6 °C every 10 s, reaching a total increase of 15.4 °C during 1 min at 10 W (24). However, because thermal cameras only measure the surface, these data alone could not confirm selective heating of the fat layer.

Probe-based thermometry enabled direct observation of temperature changes within each tissue layer. As shown in Figure 4C, the skin layer exhibited a maximum temperature rise of 4.8 °C, whereas the subcutaneous fat layer showed a peak increase of 7.5 °C-demonstrating a distinctly greater heating effect in the fat layer (21–23, 41). This pattern was consistent with the simulation results, which predicted the highest SAR and temperature rise within subcutaneous fat. Although the absolute temperature rise measured by the probes was slightly lower than that obtained from thermal imaging, this discrepancy was expected because deep-tissue heating is subject to attenuation, and the probes record local passive measurements rather than software-tracked maxima.

Overall, both qualitative infrared images and quantitative thermocouple data confirmed effective tissue heating. The subcutaneous fat layer exhibited the highest temperature elevation, closely matching simulation predictions. The skin surface showed moderate warming, while the deep-muscle region remained largely unaffected. These results clearly demonstrate that the proposed antenna achieved targeted-depth and layer-specific heating concentrated within the fat tissue.

### 3.2 Overall validation and implications for targeted subcutaneous heating

**Figure 5:**
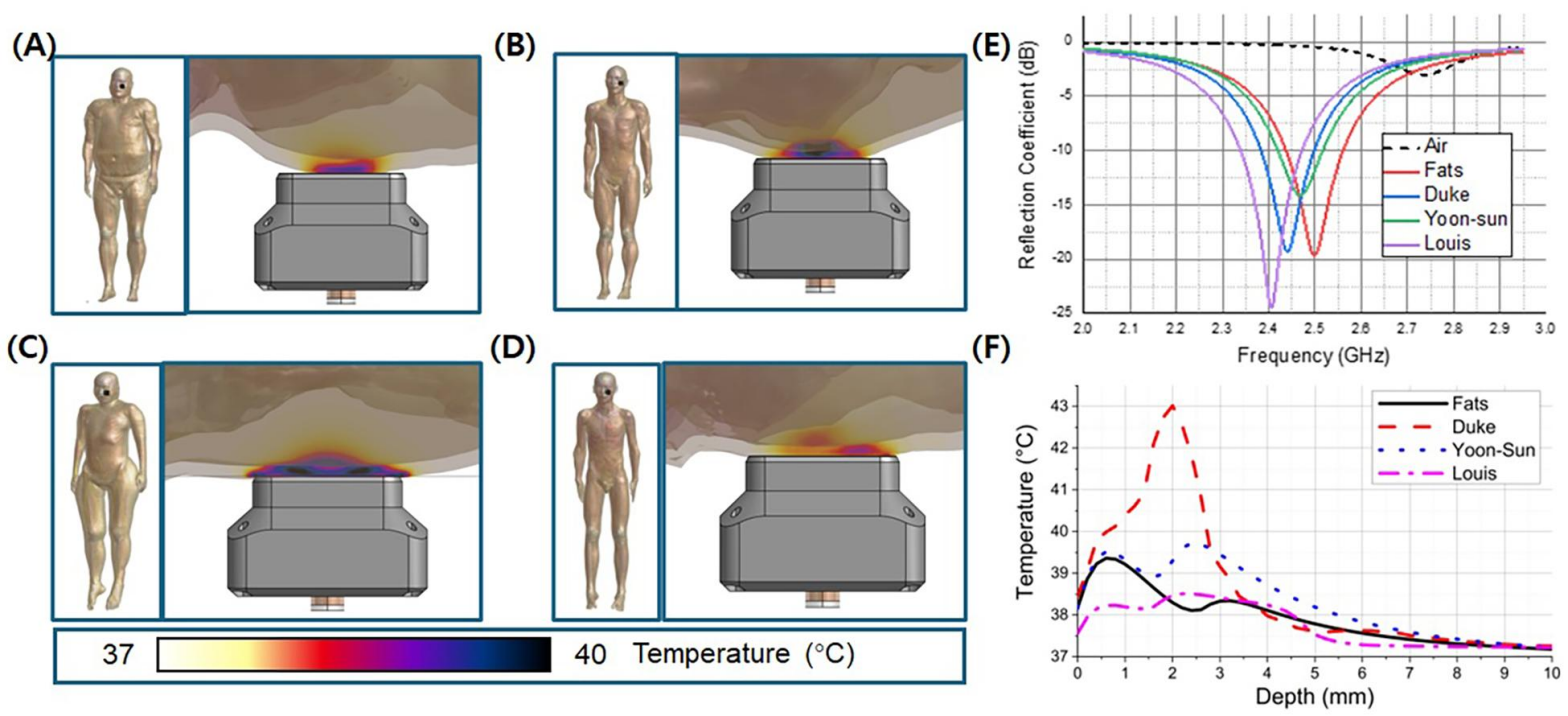
Temperature changes on Sim4Life simulation when heated to 10 W for 2 s **(A)** Fats model, **(B)** Duke model, **(C)** Yoon-Sun model, **(D)** Louis model and **(E)** S-parameter result for on-body Matching in each phantom and **(F)** Differences in temperature by model according to depth.

The combined simulation and experimental findings collectively demonstrate that the proposed antenna system reliably achieves selective heating of subcutaneous adipose tissue under non-invasive operating conditions as shown as Figure 5E. Multi-model simulations using Sim4Life confirmed that, despite anatomical variability across human phantoms as shown as Table 2, the deepest temperature rise consistently occurred within the fat layer (34, 35). As shown in Figures 5A–D, the Fats, Duke, Yoon-Sun, and Louis models exhibited temperature peaks precisely at the depth corresponding to their respective fat layers (approximately 2.3–5.2 mm beneath the surface), demonstrating consistent localization of maximal heating within adipose tissue (42). To account for the non-planar facial curvature and the potential for macroscopic air gaps in practical use, the multiphysics simulations were configured to computationally emulate soft-tissue deformation under gentle device pressure. Specifically, the flat antenna aperture was positioned to slightly overlap with the curved facial surface in the anatomical models, and higher material priority was assigned to the rigid antenna during voxelization so that overlapping skin voxels were replaced by the antenna geometry. This conformal voxel overlapping technique mimics the clinically intended application in which slight pressure flattens compliant facial soft tissue to maintain a flush, air–gap-free interface; nevertheless, severe anatomical irregularities and imperfect contact remain realistic sources of potential detuning and thermal variability. The summarized temperature-depth profiles in Figure 5F further illustrate this trend, showing that each phantom's peak temperature coincided with the boundary of the subcutaneous fat region while skin and deep muscle layers exhibited lower heating. Complementary *ex vivo* and *in vivo* measurements further validated this behavior, showing reproducible thermal responses in both biological tissue and human skin, and confirming the device's ability to induce targeted warming without excessive surface overheating.

**Table 2:** Specifications for Sim4Life phantom methods.

Name	Sex	Age [year]	Height [m]	Weight [kg]	BMI [kg/m^2^]
Fats	Male	37	1.82	119	36
Duke	Male	34	1.77	70.2	22.4
Yoon-sun	Female	26	1.52	54.6	23.6
Louis	Male	14	1.68	49.7	17.6

To assess contact safety during high-power short pulses, we quantified the applicator surface temperature under a 2 s excitation at therapeutic power. Across four anatomical models, the maximum applicator surface temperature remained below 40 °C (range: 38.2–39.5 °C at 80 W, 2 s), supporting the feasibility of brief contact operation without active cooling.

Importantly, the agreement between electromagnetic modeling, thermocouple measurements, and infrared thermography establishes a coherent verification framework that links computational predictions with physical experimental outcomes. This integrated approach provides strong evidence that the antenna can achieve controlled subcutaneous hyperthermia while maintaining surface safety, even under compact form-factor constraints. Taken together, these results confirm that the proposed antenna system fulfills its design objective of delivering reliable, depth-confined thermal stimulation that aligns with simulation-based expectations. Such capability highlights the antenna's clear potential for a wide range of non-invasive applications where controlled subdermal heating is essential, including localized fat reduction, facial skin tightening, and therapeutic hyperthermia.

These validated trends-supported by consistent fat-layer temperature peaks across Figure 5F—provide a solid empirical and computational foundation for further interpretation of the antenna's performance and for understanding the underlying mechanisms that enable targeting deep-tissue heating.

## 4 Discussion

In contrast to conventional 1–10 MHz volumetric RF systems (which deposit energy broadly and require aggressive surface cooling) and to complex phased-array applicators (which rely on active beamforming and large hardware), the proposed 2.45 GHz stacked cavity-backed metastructure achieves depth-selective heating via a passive near-field boundary mechanism. The extreme dielectric contrast at the 1.5 mm skin-fat interface causes the normal component of the electric field in adipose tissue to be amplified, effectively overcoming the skin's higher conductivity and confining SAR in the subcutaneous fat layer. This passive boundary focusing localizes energy in the target fat without active focusing or cooling, allowing a single-element applicator to heat the intended layer while sparing the skin.

Safety is quantified under realistic conditions. Using a low-loss RF-10 (ceramic-filled PTFE) dielectric stack and brief pulses (1–2 s) minimizes applicator self-heating. Finite-element thermal simulations applying 80 W for 2 s show the antenna surface temperature peaks at 38.2–39.5 °C across four human models, and experimental infrared imaging confirms the skin-surface temperature remains below ≈40 °C during operation. These results demonstrate that high-power, short bursts can safely raise fat temperature. SAR distributions are reported as un-averaged local point SAR (computed per finite-element tetrahedron and normalized to a 100 W input) to capture the sharp interface peak. A standard 1 g/10 g volume average (∼1 cm^3^) across the 1.5 mm boundary would smear this localized hot spot, so the peak point SAR is presented instead. The multiphysics thermal model solves the Pennes bioheat equation (including blood perfusion and metabolic heat from IT'IS tissue parameters) to predict realistic temperature rise.

Finally, we explicitly acknowledge limitations. As a near-field device, performance depends on applicator-skin contact. simulations assumed conformal contact via a voxel-overlap technique (slight overlap and priority voxelization) to mimic gentle tissue compression, but severe curvature or air gaps could detune the field. The *ex vivo* porcine experiments represent a worst-case (no perfusion) scenario; *in vivo* treatment would benefit from active perfusion cooling. Clinically, only surface temperature was monitored (via infrared) since direct deep-tissue thermometry is impractical. The current prototype has a fixed penetration depth; future work should include adjustable-depth designs and larger clinical trials (using a pulse-and-move protocol) to fully assess efficacy and safety.

## Data Availability

The original contributions presented in the study are included in the article/Supplementary Material, further inquiries can be directed to the corresponding authors.

## References

[1] Acar B Yilmaz T Yapar A. Optimization of microwave hyperthermia system for focused breast cancer treatment: a study using realistic digital breast phantoms. Med Phys. (2025) 52:3557–69. 10.1002/mp.17836 40270084 PMC12149706

[2] Yildiz G Farhat I Farrugia L Bonello J Zarb-Adami K Sammut CV. Comparison of microwave hyperthermia applicator designs with fora dipole and connected array. Sensors. (2023) 23:6592. 10.3390/s23146592 37514884 PMC10383607

[3] Zamay TN Zamay GS Kolovskaya OS Zukov RA Petrova MM Gargaun A. Current and prospective protein biomarkers of lung cancer. Cancers (Basel). (2017) 9:155. 10.3390/cancers9110155 29137182 PMC5704173

[4] Dewhirst MW Viglianti BL Lora-Michiels M Hanson M Hoopes PJ. Basic principles of thermal dosimetry and thermal thresholds for tissue damage from hyperthermia. Int J Hyperthermia. (2003) 19:267–94. 10.1080/0265673031000119006 12745972

[5] Chiang J Song L Abtin F Rahmat-Samii Y. Efficacy of a lung-tuned monopole antenna for microwave ablation: analytical solution and validation in a ventilator-controlled *ex vivo* porcine lung model. IEEE J Electromagn RF Microw Med Biol. (2021) 5:295–304. 10.1109/JERM.2021.3066103 35706532 PMC9191847

[6] Gongalsky M Gvindzhiliia G Tamarov K Shalygina O Pavlikov A Solovyev V. Radiofrequency hyperthermia of cancer cells enhanced by silicic acid ions released during the biodegradation of porous silicon nanowires. ACS Omega. (2019) 4:10662–9. 10.1021/acsomega.9b01030 31460163 PMC6648043

[7] Zhou J Yao D Qian Z Hou S Li L Jenkins ATA. Bacteria-responsive intelligent wound dressing: simultaneous *in situ* detection and inhibition of bacterial infection for accelerated wound healing. Biomaterials. (2018) 161:11–23. 10.1016/j.biomaterials.2018.01.024 29421548

[8] Zhang D Wen L Huang R Wang H Hu X Xing D. Mitochondrial specific photodynamic therapy by rare-earth nanoparticles mediated near-infrared graphene quantum dots. Biomaterials. (2018) 153:14–26. 10.1016/j.biomaterials.2017.10.034 29096398

[9] Dong L Gong J Wang Y He J You D Zhou Y. Chiral geometry regulates stem cell fate and activity. Biomaterials. (2019) 222:119456. 10.1016/j.biomaterials.2019.119456 31476662

[10] Guillen Fabi S. Noninvasive skin tightening: focus on new ultrasound techniques. Clin Cosmet Investig Dermatol. (2015) 8:47–52. 10.2147/CCID.S69118 25709486 PMC4327394

[11] Greene RM Green JB. Skin tightening technologies. Facial Plast Surg. (2014) 30:62–7. 10.1055/s-0033-1363756 24488639

[12] Weiss RA. Noninvasive radio frequency for skin tightening and body contouring. Semin Cutaneous Med Surg. (2013) 32:9–17.PMID: 24049924. 24049924

[13] Manna A De Forni D Bartocci M Pasculli N Poddesu B Lista F. SARS-CoV-2 inactivation in aerosol by means of radiated microwaves. Viruses. (2023) 15:1443. 10.3390/v15071443 37515131 PMC10386662

[14] Yamamoto Y Obayashi K Okano Y Satoh Y Masaki H Funasaka Y. Efficacy of thermal stimulation on wrinkle removal via the enhancement of collagen synthesis. J Derm Sci Suppl. (2006) 2:S39–49. 10.1016/j.descs.2006.08.006

[15] Rousseaux I Robson S. Body contouring and skin tightening using a unique novel multisource radiofrequency energy delivery method. J Clin Aesthet Dermatol. (2017) 10:24–9.PMID: 28458771 28458771 PMC5404777

[16] Hurwitz D Smith D. Treatment of overweight patients by radiofrequency-assisted liposuction (RFAL) for aesthetic reshaping and skin tightening. Aesthetic Plast Surg. (2012) 36:62–71. 10.1007/s00266-011-9783-z 21751063

[17] Prasad B Kim JK Kim S. Role of simulations in the treatment planning of radiofrequency hyperthermia therapy in clinics. J Oncol. (2019) 2019:1–12. 10.1155/2019/9685476 PMC6735211 31558904

[18] Shin JW Park JT Chae JB Choi JY Na JI Park KC. The efficacy of micro-insulated needle radiofrequency system for the treatment of lower eyelid fat bulging. J Dtsch Dermatol Ges. (2019) 17:149–56. 10.1111/ddg.13736 30698910

[19] Tanaka Y. Skin tightening following multisource, phase-controlled radiofrequency treatments with novel unique concentric electrodes in Asian patients. J Clin Aesthet Dermatol. (2019) 12:E58–63.PMID: 32038767 32038767 PMC7002044

[20] Franco W Kothare A Ronan SJ Grekin RC McCalmont TH. Hyperthermic injury to adipocyte cells by selective heating of subcutaneous fat with a novel radiofrequency device: feasibility studies. Lasers Surg Med. (2010) 42:361–70. 10.1002/lsm.20925 20583242

[21] Kim I Lee DM Lee YJ Shin JW Kim ES Lee H. Dual-band on-body near field antenna for measuring deep core temperature with a microwave radiometer. IEEE Access. (2022) 10:63715–22. 10.1109/ACCESS.2022.3183223

[22] Kim I Lee DM Cho MH Lee YJ Han JH Shin JW. Compact dual-band on-body near field antenna with reflector for measuring deep core temperature. IEEE Access. (2023) 11:32944–53. 10.1109/ACCESS.2023.3262997

[23] Kim I Lee DM Shin JW Lee GJ Kim ES Kim NY. Radio frequency hyperthermia system for skin tightening effect by filled waveguide aperture antenna with compact metamaterials. Front Bioeng Biotechnol. (2024) 12:1378084. 10.3389/fbioe.2024.1378084 38605987 PMC11007180

[24] Kwon TR Lee SE Kim JH Jeon YJ Jang YN Yoo KH. The effectiveness of 448-khz capacitive resistive monopolar radiofrequency for subcutaneous fat reduction in a porcine model. Med Lasers. (2019) 8:64–73. 10.25289/ML.2019.8.2.64

[25] Ouerghi K Fadlallah N Smida A Ghayoula R Fattahi J Boulejfen N. Circular antenna array design for breast cancer detection. In: 2017 Sensors Networks Smart and Emerging Technologies (SENSET) (2017). p. 1–4. 10.1109/SENSET.2017.8125016

[26] Akman OE Biringen S Waggy SB. Analysis of signal propagation in an elastic-tube flow model. Med Eng Phys. (2011) 33:660–3. 10.1016/j.medengphy.2010.12.011 21242097

[27] Pan YM Hu PF Zhang XY Zheng SY. A low-profile high-gain and wideband filtering antenna with metasurface. IEEE Trans Antennas Propag. (2016) 64:2010–6. 10.1109/TAP.2016.2535498

[28] Mahmoud KR Montaser AM. Design of multiresonance flexible antenna array applicator for breast cancer hyperthermia treatment. IEEE Access. (2022) 10:93338–52. 10.1109/ACCESS.2022.3203431

[29] Chakaravarthi G Arunachalam K. Design and characterisation of miniaturised cavity-backed patch antenna for microwave hyperthermia. Int J Hyperthermia. (2015) 31:737–48. 10.3109/02656736.2015.1068957 26365603

[30] Mishra A Khan A Dubey SK. Design and optimization of a miniaturized antenna for targeted hyperthermia in tumor therapy. Am J Electromagn Appl. (2025) 13:1–7. 10.11648/j.ajea.20241301.11

[31] Zhu S Liu H Wen P. A new method for achieving miniaturization and gain enhancement of cicaldi antenna array based on anisotropic metasurface. IEEE Trans Antennas Propag. (2019) 67:1952–6. 10.1109/TAP.2019.2891220

[32] Numan AB Sharawi MS. Extraction of material parameters for metamaterials using a full-wave simulator [education column]. IEEE Antennas Propag Mag. (2013) 55:202–11. 10.1109/MAP.2013.6735515

[33] Fernandez M Espinosa HG Thiel DV Arrinda A. Wearable slot antenna at 2.45 GHz for off-body radiation: analysis of efficiency, frequency shift, and body absorption. Bioelectromagnetics. (2018) 39:25–34. 10.1002/bem.22081 28898435

[34] Singh S Singh SP Singh D. Compact conformal metasurface antenna for hyperthermia applications. J Electromagn Waves Appl. (2023) 37:950–65. 10.1080/09205071.2023.2216394

[35] Gosselin MC Neufeld E Moser H Huber E Farcito S Gerber L. Development of a new generation of high-resolution anatomical models for medical device evaluation: the virtual population 3.0. Phys Med Biol. (2014) 59:5287. 10.1088/0031-9155/59/18/5287 25144615

[36] Ortega-Palacios R Trujillo-Romero CJ Cepeda Rubio MFJ Vera A Leija L Reyes JL. Feasibility of using a novel 2.45 GHz double short distance slot coaxial antenna for minimally invasive cancer breast microwave ablation therapy: computational model, phantom, and *in vivo* swine experimentation. J Healthc Eng. (2018) 2018:5806753. 10.1155/2018/5806753 29854360 PMC5964617

[37] Xiong H Xie JY Liu YJ Wang BX Xiao DP Zhang HQ. Microwave hyperthermia technology based on near-field focused metasurfaces: design and implementation. Adv Funct Mater. (2025) 35:2411842. 10.1002/adfm.202411842

[38] Abdul-Al M Amar ASI Elfergani I Littlehales R Ojaroudi Parchin N Al-Yasir Y. Wireless electromagnetic radiation assessment based on the specific absorption rate (sar); A review case study. Electronics (Basel). (2022) 11:511. 10.3390/electronics11040511

[39] Karacolak T Cooper R Unlu ES Topsakal E. Dielectric properties of porcine skin tissue and *in vivo* testing of implantable antennas using pigs as model animals. IEEE Antennas Wirel Propag Lett. (2012) 11:1686–9. 10.1109/LAWP.2013.2241722

[40] Wang L Bao X Wang Y Yan S Zhang A. A minimally invasive microwave ablation antenna with highly localized ablation zone. IEEE Antennas Wirel Propag Lett. (2022) 21:1587–91. 10.1109/LAWP.2022.3174872

[41] Burns JA. Thermage: monopolar radiofrequency. Aesthetic Surg J. (2005) 25:638–42. 10.1016/j.asj.2005.09.017 19338872

[42] Hasgall PA Di Gennaro F Baumgartner C Neufeld E Gosselin MC Payne D. IT'IS database for thermal and electromagnetic parameters of biological tissues, Version 4.1 (2022).

[43] IEEE Recommended practice for measurements and computations of electric, magnetic, and electromagnetic fields with respect to human exposure to such fields, 0 Hz to 300 GHz. In: IEEE Std C95.3-2021 (Recision of IEEE Std C95.3-2002 and IEEE Std C95.3.1-2010) (2021). p. 1–240.

